# A Lithium‐Ion Pump Based on Piezoelectric Effect for Improved Rechargeability of Lithium Metal Anode

**DOI:** 10.1002/advs.201901120

**Published:** 2019-09-17

**Authors:** Jingwei Xiang, Zexiao Cheng, Ying Zhao, Bao Zhang, Lixia Yuan, Yue Shen, Zezhou Guo, Yi Zhang, Jianjun Jiang, Yunhui Huang

**Affiliations:** ^1^ State Key Laboratory of Material Processing and Die & Mold Technology School of Materials Science and Engineering Huazhong University of Science and Technology Wuhan 430074 China; ^2^ Department of Engineering Cambridge University Trumpington St. CB2 1PZ Cambridge UK; ^3^ School of Optical and Electronic Information Huazhong University of Science and Technology Wuhan 430074 China

**Keywords:** dendrite‐free, lithium metal anode, lithium‐ion pump, lithium‐sulfur batteries, piezoelectric effect

## Abstract

Lithium metal is widely studied as the “crown jewel” of potential anode materials due to its high specific capacity and low redox potential. Unfortunately, the Li dendrite growth limits its commercialization. Previous research has revealed that the uniform Li‐ion flux on electrode surface plays a vital role in achieving homogeneous Li deposition. In this work, a new strategy is developed by introducing a multifunctional Li‐ion pump to improve the homogenous distribution of Li ions. Via coating a β‐phase of poly(vinylidene fluoride) (β‐PF) film on Cu foil (Cu@β‐PF), a piezoelectric potential across such film is established near the electrode surface because of its piezoelectric property, which serves as a driving force to regulate the migration of Li ions across the film. As a result, uniform Li‐ion distribution is attained, and the Cu@β‐PF shows coulombic efficiency around 99% throughout 200 cycles. Meanwhile, the lithium‐sulfur full cell paired with Li‐Cu@β‐PF anode exhibits excellent performance. This facile strategy via regulating the Li‐ion migration provides a new perspective for safe and reliable Li metal anode.

## Graphical abstract

**Via coating a piezoelectric film on Cu/Li foil, a piezoelectric potential** is established near the electrode surface, which serves as a driving force to regulate the migration of Li ions. As a result, uniform Li‐ion distribution is attained and the Cu@β‐PF shows coulombic efficiency around 99% throughout 200 cycles. Meanwhile, the lithium‐sulfur full cell also exhibits excellent performance.

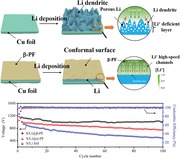

The ever‐increasing demands for advanced energy storage equipment thrust the research on high‐energy‐density lithium‐ion batteries (LIBs) into a climax in recent years.^1^, ^2^ However, the inherent limited capacity of existed LIBs cannot satisfy the key market's demands for next‐generation energy storage, such as the electric vehicles.^3^, ^4^ Therefore, it becomes more urgent to explore new materials or even new electrochemistry beyond LIBs than at any time in the past. Among all possible candidates, lithium (Li) metal anode, which owns the lowest negative electrochemical potential (−3.04 V vs the stand hydrogen electrode) and the lightest density (0.534 g cm^−3^), is receiving more and more attention around the world as the “Holy Grail” anode material for lithium metal batteries (LMBs), including lithium‐oxygen (Li‐O_2_) batteries, lithium‐sulfur (Li‐S) batteries, and so on.^5^, ^6^, ^7^

Unfortunately, the commercial application of Li metal anode still has a long way off before the formidable challenges of safety and cyclability being completely overcome. Similar to some other metal electrodes, such as Zn, Ni, Na, etc., Li metal anode also encounters the dendrite growth issue during the electrodeposition process.^3^ During the repeated nonuniform deposition/dissolution of Li ions, the Li dendrite will continue to grow until it contacts the cathode, thus causing internal short‐circuit and thermal runaway of the cell, which might result in the burning of the batteries and cause serious safety issues.^8^, ^9^ In addition, due to the thermodynamic instability of Li metal in most nonaqueous solvents, a solid electrolyte interphase (SEI), which is electrically insulating and ionically conductive, will spontaneously form on the Li metal surface once the Li metal comes into contact with nonaqueous electrolytes, thus effectively separating the electrolytes from Li metal and preventing the consuming reactions.^10^, ^11^ Nevertheless, the native SEI is too fragile to accommodate the large volume change of the electrode or Li dendrite growth. What is worse, once the fresh Li is exposed to the electrolyte, new SEI will form constantly, which will keep on consuming the electrolyte and Li metal. Therefore, realizing dendrite‐free Li metal anode and constructing more flexible interface are crucial for the commercialization of Li metal anode.^12^ Fortunately, great achievement has been made in the development of Li metal anode after years' efforts. 3D conductive Li hosts, such as 3D graphene,^13^, ^14^, ^15^ porous copper,^16^, ^17^ and so on, are designed by many groups to decrease the actual current density and regulate the Li‐ion deposition, thus effectively suppressing the Li dendrite growth. Some electrolyte additives like Cs^+^ 18 and Li^+^ halides^19^ have also been proven very useful to strengthen the SEI or improve the homogenous Li‐ion deposition/dissolution. In addition, employing solid‐state electrolytes^20^, ^21^ and modifying the Li metal surface^22^, ^23^ are effective strategies to lower the reaction activity between Li anode and electrolytes, thus greatly improving the interfacial stability and delaying the Li dendrite growth. All these strategies have positive effects on suppressing Li dendrite growth or enhancing the coulombic efficiency (CE). However, convection always exists in the mass transfer process on the surface of Li anode, which inevitably causes the nonuniform Li‐ion flux and hence the nonuniform Li deposition after long‐term cycling.^2^ As shown in **Figure** **1**a, the light‐colored regions correspond to the Li‐ion deficiency. Without any treatment, the nonuniform Li deposition finally results in Li dendrite growth and porous Li electrode on the Cu foil. Therefore, pursuing homogenous distribution of Li ions near the surface of Li anode is of crucial significance to realize the practical applications of Li metal batteries.

**Figure 1:**
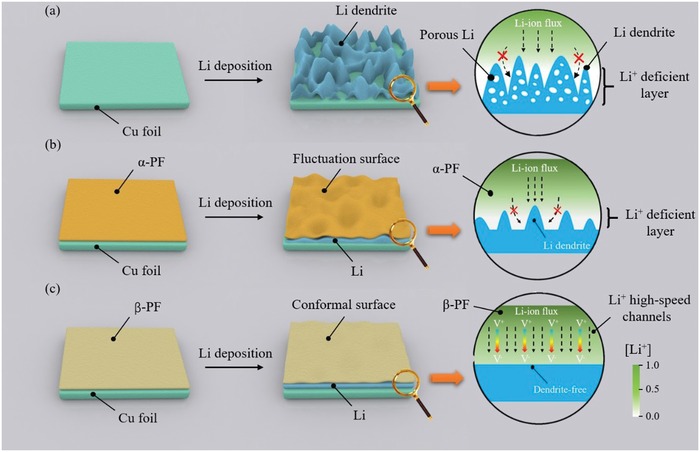
Schematic illustration of Li deposition on Cu foil with different treatments: a) without protection, leading to the formation of porous Li dendrite and Li‐ion deficient layer on the Cu foil after deposition; b) with modification of α‐PF film, forming fluctuation surface and Li‐ion‐deficient layer on the Cu foil after deposition; c) with β‐PF film modification, serving as the Li‐ion pump to regulate the uniform distribution of Li ions.

Recently, polymeric coating has been widely used in the protection of Li metal anode as an artificial SEI because of their high modulus.^24^, ^25^ However, for a Cu foil current collector with conventional α‐phase of poly(vinylidene fluoride) (α‐PF) modification, the Li‐ion migration is much slower than the charge transfer process at the surface of Li anode during the Li deposition, which leads to the formation of a Li‐ion‐deficient layer near the electrode surface and the growth of Li dendrite (Figure 1b). To solve this issue, we try to introduce a multifunctional Li‐ion pump based on ferroelectric material, which has already been employed in Li‐ion batteries and supercapacitors to improve the electrochemical performance and migration of Li ions by creating an internal electric field. When compress stress is applied on the piezoelectric polyvinylidene fluoride (PVDF) in assembled coin cell, a piezoelectric field with a direction pointing from the cathode to the anode is generated. Then Li ions are driven by the piezoelectric field and migrate from cathode to anode in the electrolyte through the PVDF film. What's more, the thickness of Li metal anode will ever increase during the deposition of Li ions, which lead to enhanced cell pressure and piezoelectric field.^26^, ^27^, ^28^, ^29^, ^30^ The Li‐ion pump can not only effectively boost the homogeneous deposition of Li ions at the surface of Li anode but also improve the interface stability of the Li metal/electrolyte. Here, β‐phase of poly(vinylidene fluoride) (β‐PF) is chosen to build the Li‐ion pump, which has good piezoelectric property and excellent processability.^30^, ^31^ The β‐PF film coated on the Cu foil establishes a piezoelectric potential under pressure across itself near the electrode surface, which acts as not only an artificial SEI but also a driving force to accelerate the migration of Li ions across the film near the electrode surface (Figure 1c). Therefore, with β‐PF modification, high‐speed channels for Li ions have been formed across the coating film, thus eliminating the Li‐ion‐deficient layer and effectively improving the homogeneous Li‐ion deposition. As a result, the CE of this modified Li metal anode is greatly enhanced, which retains to ≈99% even after 200 cycles. When paired with sulfur cathode, the full cell performs very well with a capacity over 1000 mAh g^−1^ after 100 cycles.

The β‐PF film was prepared by spreading a solution of PF in N,N‐dimethyl formamide (DMF) on a Cu foil, followed by an evaporation at 60 °C to ensure the isothermal crystallization of PF during which the phase of PF changes from α to β successfully.^31^, ^32^, ^33^ As shown in Figure S2 (Supporting Information), β‐PF film with uniform morphology is obtained and the thickness of this protective layer is ≈5 µm. The polarization–electric field loops of the PF film at a constant frequency of 100 Hz and gradually increased voltages are as shown in Figure S3 (Supporting Information). For comparison, the conventional α‐PF film was produced through the same solution crystallization process but drying at high temperature. Fourier‐transformed infrared (FTIR) spectra and X‐ray diffraction (XRD) are used to verify the crystalline phase of the films. As shown in **Figure** **2**a,b, the β‐PF film shows the characteristic peaks at 510 and 840 cm^−1^ in the FTIR spectra and the diffraction peak at 2θ = 20.3° in the XRD spectra, corresponding to the predominantly β‐crystalline phase.^31^, ^34^, ^35^ For the α‐PF film, it shows the FTIR peaks located at 532, 614, and 764 cm^−1^, which are the typical signals of the α phase. The resulted XRD peaks located at 17.7°, 18.2°, and 19.9° also confirm the presence of α phase.^31^, ^32^, ^33^

**Figure 2:**
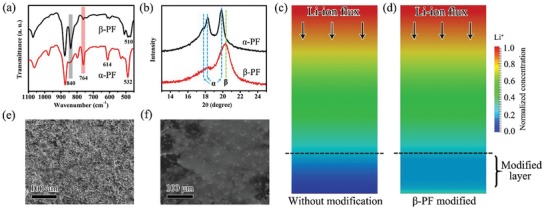
Properties of PF film coatings and the effects of β‐PF film on the behavior of Li ions. a) FTIR spectra and b) XRD patterns of α‐PF and β‐PF films; c,d) the simulation of the distribution of Li ions without modification and with β‐PF modified; e,f) surface SEM images of Li without modification and with β‐PF modified after 50 cycles.

For better understanding how the β‐PF film influences the behavior of Li ions in the electrolyte, a model is built to analyze the distribution of Li ions in the electrolyte (more details are given in the Supporting Information). The distribution of Li‐ion concentration in traditional liquid organic electrolyte is shown in Figure 2c. Due to the nonuniform Li‐ion flux on the electrode surface itself and the slow Li‐ion migration in the electrolyte, the concentration distribution of Li ions is inhomogeneous in the direction paralleling to the electrode in electrolyte, which ultimately results in the appearance of Li‐ion‐deficient layer near the anode. Therefore, it is necessary to compensate the Li ions near the electrode to remove the Li‐ion‐deficient layer. In contrast, due to the piezoelectric potential established by the β‐PF film, high‐speed channels have been built for Li ions across the β‐PF film, which can drive the fast migration of Li ions across the film with a direction from cathode to anode, thus contributing to the uniform distribution of Li ions near the electrode. As shown in Figure 2d, with β‐PF film modification, the Li‐ion‐deficient layer is removed and Li ions uniformly distribute near the electrode.

To verify the reliability of the simulation, a commercial Li foil modified by the β‐PF film (Li@β‐PF) and a Li foil without modification were used as the working electrodes for the test cells. For the Li foil without modification, the surface of Li foil is covered with quantities of Li dendrites and becomes porous after 50 cycles at 1 mA cm^−2^ for 2 mAh cm^−2^ (Figure 2e), which is ascribed to the accumulation of uneven deposition of Li ions. Obviously, the SEI formed on the surface of Li foil is too weak to bear the huge volume change of the Li dendrites during cycling, thus resulting in an unstable Li metal/electrolyte interface and side reactions. In contrast, as shown in Figure 2f, after peeling off the β‐PF film coating on the Li foil, the surface becomes relatively smooth and no obvious Li dendrite can be found after cycling, which provides a powerful evidence that the Li dendrite has been effectively suppressed by the β‐PF film. According to the optical photographs of Li foil (Figure S4a,b, Supporting Information) and Li@β‐PF (Figure S4c,d, Supporting Information) before and after cycling, the surface of Li@β‐PF still remains smoother with metallic luster after cycling when compared with Li foil, revealing that Li dendritic growth has been effectively suppressed. To further confirm the piezoelectric property of β‐PF after cycles, the FTIR spectra were conducted to verify the crystalline phase of these films after cycles. As shown in Figure S5 (Supporting Information), it shows the FTIR peak located at 840 cm^−1^, which are the typical signals of the β phase. The result indicates that this coating film can still retain β phase even after cycles, thus keeping the piezoelectric property.

The behaviors of Li‐ion depositing on different treated commercial Cu foils were further investigated to check the effect of piezoelectric potential created by the β‐PF film on the protection of Li anode. **Figure** **3** shows the behaviors of the working electrodes of bare Cu foil, the Cu foils with α‐PF film (Cu@α‐PF), and the β‐PF film. For the bare Cu foil (Figure 3a–c), the surface is covered with tremendous Li dendrites after 50 cycles with 4 mAh cm^−2^ Li deposition. According to the cross‐section scanning electron microscope (SEM) image, a loose and porous morphology with a large volume change can be clearly observed (the thickness of deposited Li is ≈45 µm), which should be caused by the growth of Li dendrite as well as the continuous side reactions between the electrolyte and deposited Li. Different from the untreated Cu foil, the volume expansion of the deposited Li has been effectively limited (≈26 µm) by the artificial SEI of the coated α‐PF film (Figure 3d–f). However, due to the limited Li‐ion migration rate in the electrolyte, the inhomogeneous distribution of Li ions still becomes even worse during long cycles, thus resulting in Li dendrite growth and rough surface of the electrode (Figure 3e,f). Therefore, a simple physical barrier layer cannot achieve a uniform Li deposition morphology during long cycles. In sharp contrast, as shown in Figure 3g, the piezoelectric potential established by the β‐PF film can be regarded as a powerful force to drive the migration of Li ions toward the electrode. Many Li‐ion high‐speed channels have been built up across the β‐PF film, leading to the Li‐ion compensation of near the electrode and more uniform distribution of Li ions. Therefore, this multifunctional β‐PF film not only acts as a robust artificial SEI to suppress the growth of Li dendrite but also serves as an ion pump to facilitate the uniform distribution of Li ions. With β‐PF film modified on the Cu foil (Cu@β‐PF), it can be clearly seen that the surface of the anode is still very smooth even after 50 cycles (Figure 3h). As shown in the cross‐section SEM image (Figure 3i), the β‐PF film remains stable, and the deposited Li below the β‐PF film is much denser (≈22 µm) than that on Cu foil or Cu@α‐PF, which should be attributed to the greatly improved distribution of Li ions. Therefore, compared to the bare Cu foil or Cu@α‐PF, the piezoelectric potential created by the β‐PF film plays a vital role in realizing the uniform deposition of Li ions.

**Figure 3:**
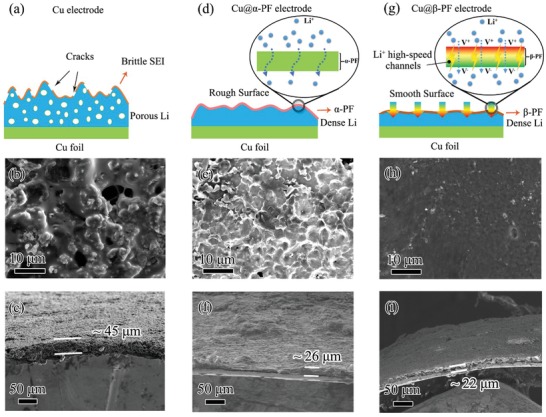
Characterization of Cu, Cu@α‐PF, and Cu@β‐PF electrodes after cycling. a,d,g) Schematic illustration of Cu, Cu@α‐PF, and Cu@β‐PF after cycles; b,e,h) surface SEM images of Cu, Cu@α‐PF, and Cu@β‐PF after 50 cycles with 4 mAh cm^−2^ Li deposition; c,f,i) cross‐sectional SEM images of Cu, Cu@α‐PF, and Cu@β‐PF after 50 cycles with 4 mAh cm^−2^ Li deposition.

To further investigate what effect β‐PF film have on the transportation of Li ions, the ionic conductivities of different samples in traditional 1 m LiTFSI/DOL+DME electrolyte have been tested (**Figure** **4**a–d; Figures S6 and S7, Supporting Information). The ionic conductivity of β‐PF@PP/PE (the PP/PE separator modified with β‐PF film) is about 2.65 mS cm^−1^, which is relatively higher than that of PP/PE without modification (2.17 mS cm^−1^). And the Li^+^ transference number of β‐PF@PP/PE (0.53) is also higher than that of PP/PE (0.19). However, when the piezoelectric polarization of β‐PF is reversed (re‐β‐PF@PP/PE), the Li^+^ transference number of re‐β‐PF@PP/PE (0.11) becomes even lower than that of PP/PE (0.19), which proves that the reversed piezoelectric polarization can drive the migration of Li ions in the opposite direction. As a result, the β‐PF coating layer can effectively drive the fast migration of Li ions. Meanwhile, we assembled half cells to test the CE values. In general, the CE values are measured based on the ratio of the capacity of dissolved Li to that of the deposited Li in each cycle. In Figure 4e, without any protection, the CE of the Li anode with bare Cu foil as current collector drops quickly at a current density of 2 mA cm^−2^ for 2 mAh cm^−2^. This is because the Li dendrite grows seriously. The Cu@α‐PF anode shows an increased CE than the Li‐Cu anode, and the stability of CE after long cycles is also improved. However, the situations of Cu foil and Cu@α‐PF become worse as the current density and the Li deposition capacity increase (Figure 4f). In striking contrast, with the β‐PF film modification, the CE of Cu@β‐PF can be stabilized up to ≈99% at 2 mA cm^−2^ for 2 mAh cm^−2^ without decay even after 200 long‐term cycles (Figure 4e). What's more, when the current densities increase to 4 mA cm^−2^ (Figure 4f), the CE of Cu@β‐PF can still keep around 99%, which means that the growth of Li dendrite can be effectively suppressed during long cycles.

**Figure 4:**
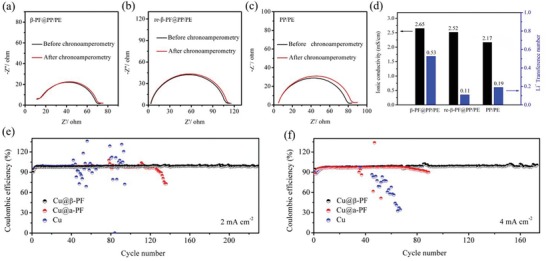
a–c) Nyquist plots of traditional 1 m LiTFSI/DOL+DME electrolyte with β‐PF@PP/PE re‐β‐PF@PP/PE, and PP/PE, and d) Li^+^ transference number and ionic conductivity of β‐PF@PP/PE, re‐β‐PF@PP/PE, and PP/PE, e,f) Coulombic efficiency of Cu, Cu@α‐PF, and Cu@β‐PF electrodes at current densities of 2 mA cm^−2^ for 2 mAh cm^−2^ and 4 mA cm^−2^ for 2 mAh cm^−2^.

The interfacial resistance of different samples before and after cycles is also studied by the electrochemical impedance spectroscopy (EIS). As shown in **Figure** **5**, it is the corresponding Nyquist plots of the symmetric Li (Figure 5a), Li@α‐PF (Figure 5b), and Li@β‐PF (Figure 5c) batteries. Compared with symmetric Li and Li@α‐PF batteries, the interface resistance of symmetric Li@β‐PF batteries is lower and more stable after 20 cycles. In addition, we further used the symmetric Li@β‐PF | Li@β‐PF, Li@α‐PF | Li@α‐PF, and Li | Li cells to evaluate the cycle stability. From Figure 5d, we can find that the overpotential of Li@β‐PF remains stable even after 900 h cycles at 2 mA cm^−2^ for 4 mAh cm^−2^, indicative of uniform Li deposition morphology and stable interface. Compared to Li@β‐PF, the overpotential of Li foil and Li@α‐PF (Figure S8, Supporting Information) is stable during the inception phase, but it increases sharply after long cycling. In order to test the feasibility of the Li@β‐PF anode, full cells paired with S cathode were assembled. For the S/Li full cell, the soluble polysulfide intermediates generated from the cathode may shuttle to Li metal anode, which will influence the stability of Li anode and lead to the fast capacity fading. Therefore, the capacity of S/Li full cell drops quickly to less than 500 mAh g^−1^ after 100 cycles (Figure 5e), which loss rate of capacity is as high as 57.5%. Compared with the S/Li full cell, the S/Li@α‐PF full cell shows better electrochemical performance. However, after 100 cycles, the capacity only remains ≈800 mAh g^−1^ (Figure 5e). Fortunately, with β‐PF film modification, the capacity can remain at ≈1000 mAh g^−1^ after 100 cycles. And according to the SEM images and energy‐dispersive X‐ray spectroscopy (EDS) results of Li metal anodes after cycling (Figure S9, Supporting Information), with β‐PF modification, the surface of the Li metal anode is relatively smoother and the signal of sulfur element on the surface is weaker when compared with that of Li metal anode without protection. Furthermore, the rate performances of S/Li@β‐PF have also been greatly improved (Figure S10, Supporting Information). Therefore, the β‐PF coating not only effectively suppresses the growth of Li dendrite but also blocks the diffusion of the soluble polysulfide intermediates.

**Figure 5:**
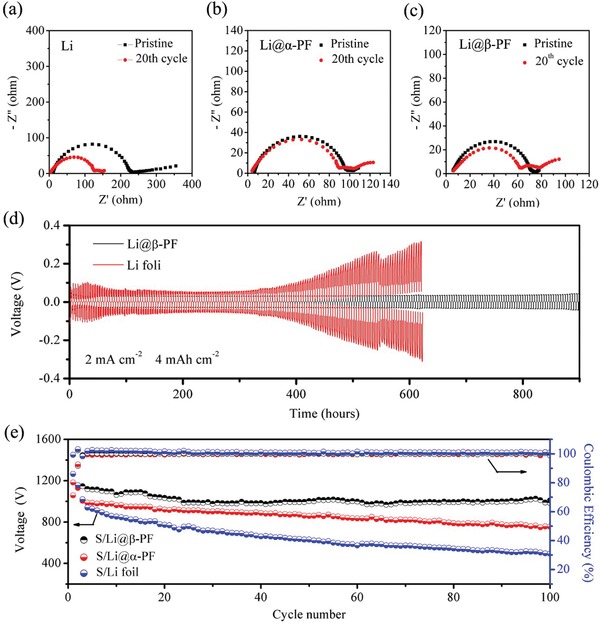
Electrochemical performances of Li@β‐PF cells and S/Li@β‐PF full cells: a–c) Nyquist plots of symmetric Li, Li@α‐PF, and Li@β‐PF batteries before and after cycles; d) voltage–time curves of symmetric Li@β‐PF | Li@β‐PF and Li | Li cells at 2 mA cm^−2^ for 4 mAh cm^−2^; e) cycle performances of Li‐S full cells with Li foil (blue), Li@α‐PF (red), and Li@β‐PF (black) as anodes at a current density of 0.2 C.

In conclusion, uniform Li‐ion flux on the electrode surface plays an important role in achieving homogeneous Li deposition. However, the distribution of Li ions near the electrode is inherently nonuniform due to convective mass transfer and slow Li‐ion migration, resulting in the growth of Li dendrite. Based on these concerns, we propose a new strategy by building up a multifunctional Li‐ion pump via a piezoelectric β‐PF layer on the surface of the anode to guide the uniform distribution of Li ions. The β‐PF film modification on the electrode surface creates a piezoelectric potential and hence contributes to drive the migration of Li ions toward the electrode. The high‐speed Li‐ion channels formed across the film can effectively promote the homogeneous deposition of Li ions near the electrode and achieve a uniform Li deposition. As a result, the stability and safety have been greatly improved. The CE of Cu@β‐PF reaches ≈99% at 2 mA cm^−2^ and still remains stable even after 200 cycles. When the current density increases to 4 mA cm^−2^, the CE is stabilized at 99% after 160 cycles. What's more, Li‐S full cell paired with Li@β‐PF exhibits excellent performance with the capacity of ≈1000 mAh g^−1^ even after 100 cycles, demonstrating the prospect of Li@β‐PF as the anode for the high‐performance Li‐S full cell. We believe that the strategy proposed here will provide a new train of thought for the practical application of Li metal anode in the high‐energy‐density systems such as Li‐S batteries.

## Conflict of Interest

The authors declare no conflict of interest.

## Supporting information

Supplementary
