# Supplementary material for: A Lithium‐Ion Pump Based on Piezoelectric Effect for Improved Rechargeability of Lithium Metal Anode
Source: Adv Sci (Weinh). 2019 Sep 17;6(22):1901120. doi: 10.1002/advs.201901120 (PMC6864497; doi:10.1002/advs.201901120)
Supplement: Supplementary file 1 — Supplementary [file ADVS-6-1901120-s001.pdf]

Copyright WILEY-VCH Verlag GmbH & Co. KGaA, 69469 Weinheim, Germany, 2019.

ADVANCED SCIENCE Open Access

Supporting Information

for *Adv. Sci.,* DOI: 10.1002/advs.201901120

A Lithium-Ion Pump Based on Piezoelectric Effect for Improved Rechargeability of Lithium Metal Anode

Jingwei Xiang, Zexiao Cheng, Ying Zhao, Bao Zhang, Lixia Yuan, Yue Shen, Zezhou Guo, Yi Zhang, Jianjun Jiang, and Yunhui Huang**

Copyright WILEY-VCH Verlag GmbH & Co. KGaA, 69469 Weinheim, Germany, 2019.

Supporting Information

A Lithium-Ion Pump Based on Piezoelectric Effect for Improved Rechargeability of Lithium Metal Anode

Jingwei Xiang, Zexiao Cheng, Ying Zhao, Bao Zhang, Lixia Yuan, Yue Shen, Zezhou Guo, Yi Zhang, Jianjun Jiang and Yunhui Huang**

Experimental Section

Preparation of the α-PF and β-PF modified electrodes: Firstly, polyvinylidene difluoride (PVDF) was dissolved in N, N-Dimethylformamide (DMF) at 10 wt% and the dispersion was magnetic stirred for overnight. Then, the PVDF solution was spread on a Cu foil with a doctor blade, followed by drying at 60 ℃ for 120 min to ensure the removal of the DMF and the isothermal crystallization of β-PF. The free-standing film after polarization can be easily separated from the substrate. In contrast, the α-PF modified electrodes were obtained by coating the PVDF solution on a Cu foil with drying at 120 ℃.

Characterizations: The crystalline phase of PVDF films were analyzed by the FTIR (Bruker Vertex 70 FTIR spectrometer) and XRD (PANalytical X′pert PRO-DY2198, Holland) with Cu Kα radiation. The morphology of the samples was characterized with a field-emission SEM (SIRION200). Ferroelectric hysteresis loops of the PVDF film was measured on a ferroelectric test module (TF2000 analyzer; axi ACCT, Germany) at a constant frequency of 100 Hz and gradually increased voltages.

Electrochemical Measurements: The galvanostatic charge/discharge experiments were measured by using the CR 2032-type coin cells. These cells were assembled in an Ar-filled glovebox with Cu, Cu@α-PF, Cu@β-PF, Li, Li@α-PF and Li@β-PF as the work electrodes and Li foils as the counter electrodes. The electrolyte adopted was composed of 1 mol L^{-1} lithium bis(trifluoromethanesulfonyl)imide (LiTFSI) solution in 1,3-dioxolane (DOL) and dimethoxymethane (DME) with 1 wt% $LiNO_3$ as additives. The ionic conductivity was tested by AC impedance measurements by using stainless steel | stainless steel coin cells and the Li^+ transference number was tested by combing the chronoamperometry and the EIS by using Li | Li coin cell. For the Li-S full cell test, the S cathode was prepared from the slurry by mixing the commercial S, the carbon black and the PVDF in a weight ratio of 6:3:1. The mass loading of S was ~2 mg cm^{-2}. The S cathode was paired with Li, Li@α-PF and Li@β-PF as the anode, separately.

Simulation of Li-ion distribution in the electrolyte

We consider a model as shown in Figure S1. The current density i in the electrolyte and PVDF can be expressed:

$$i = -D_{\text{eff}}\text{F}\frac{\partial c}{\partial x} \quad \text{in Electrolyte (1)}$$

$$i = -D_{\text{eff}}\text{F}\frac{\partial c}{\partial x} + \frac{D_{\text{eff}}F^2}{\text{RT}}cE_{\text{piezo}} \quad \text{in PVDF (2)}$$

where c is the lithium-ion molar concentration. D_{eff}, F are effective diffusivity, Faraday constant and effective conductivity in the electrolyte, respectively. E_{piezo} is the piezoelectric field generated inside the PVDF. The piezoelectric parameter g_{33} is taken to be -0.15VmN^{-1}[a]. The electric field generated in PVDF under the pressure of 0.5MPa is

$$E_{\text{piezo}} = g_{33}\text{p} = 0.15\text{Vm}N^{-1} \times 0.5\text{MPa} = 75\text{kVm}^{-1}$$

The other parameters are listed in Table 1.

D_{eff}	3×10^{-10} m^{2}s^{-1}
F	96485 sAmol^{-1}
R	8.314 Jmol^{-1}K^{-1}
T	300 K
l_1	25 µm
l_2	29 µm
i	1 mAcm^{-2}

Table 1: Simulation parameters

For the boundary condition, we assume the concentration on the surface of the anode to be zero when PVDF in Figure S1 is absent. The result is shown in manuscript Figure 2.

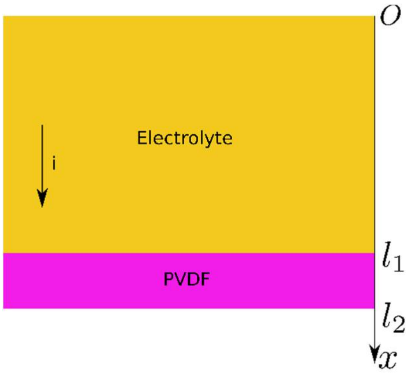

Figure S1. Schematic of the simulation domain.

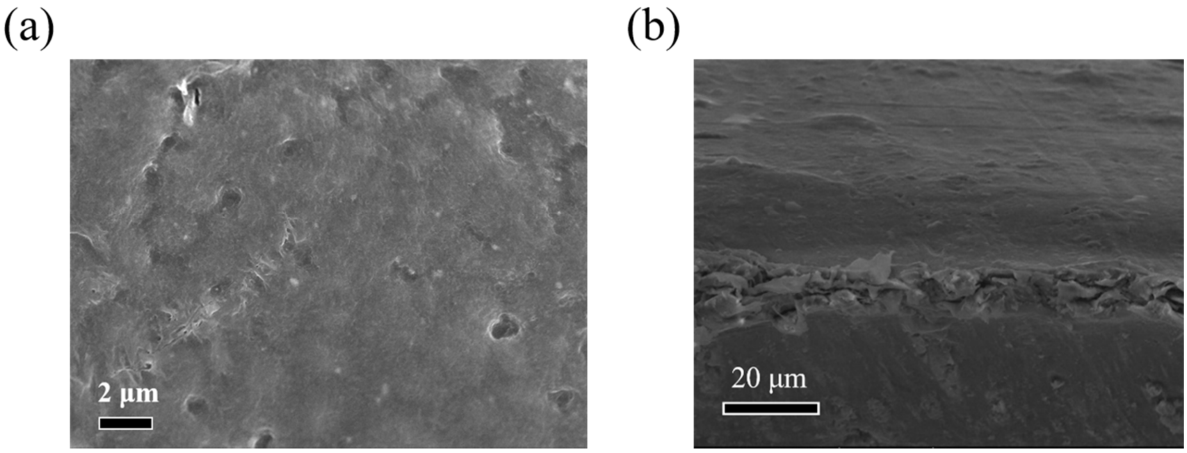

Figure S2. The surface (a) and cross-section (b) SEM images of β-PF film.

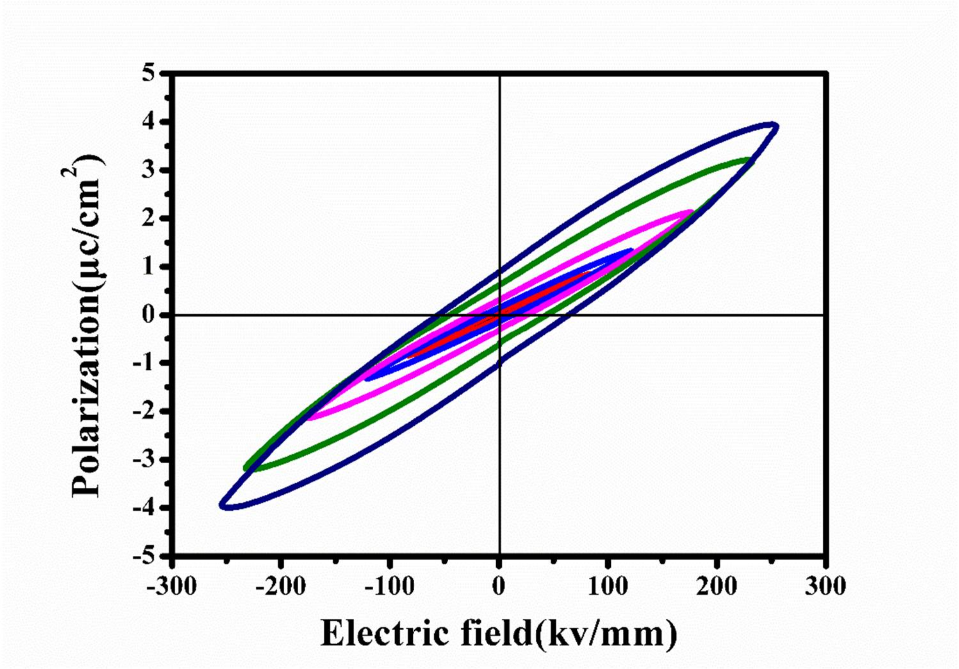

Figure S3. Polarization-electric field loops of the PF film at a constant frequency of 100 Hz.

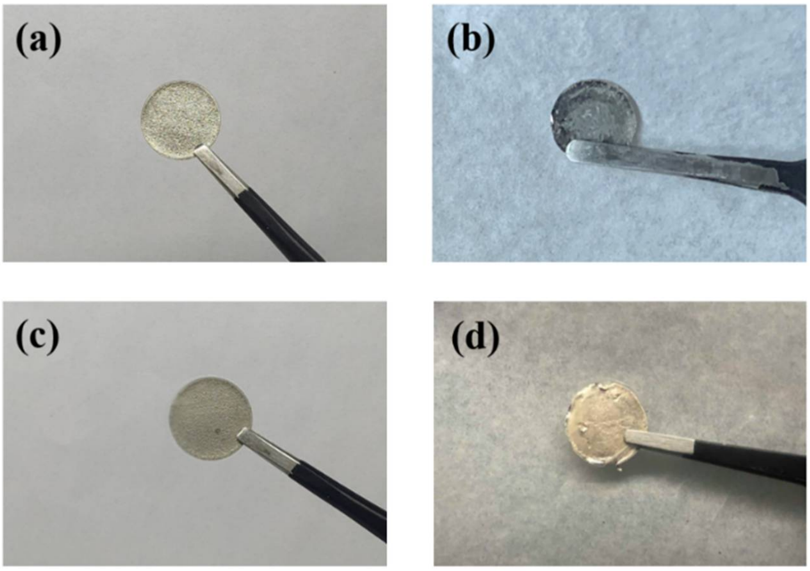

Figure S4. The optical photographs of Li metal before with/without β-PF film modified before and after cycling. a, b) the optical photographs of Li metal before (a) and after (b) cycling, c, d) the optical photographs of Li@β-PF before (c) and after (d) cycling.

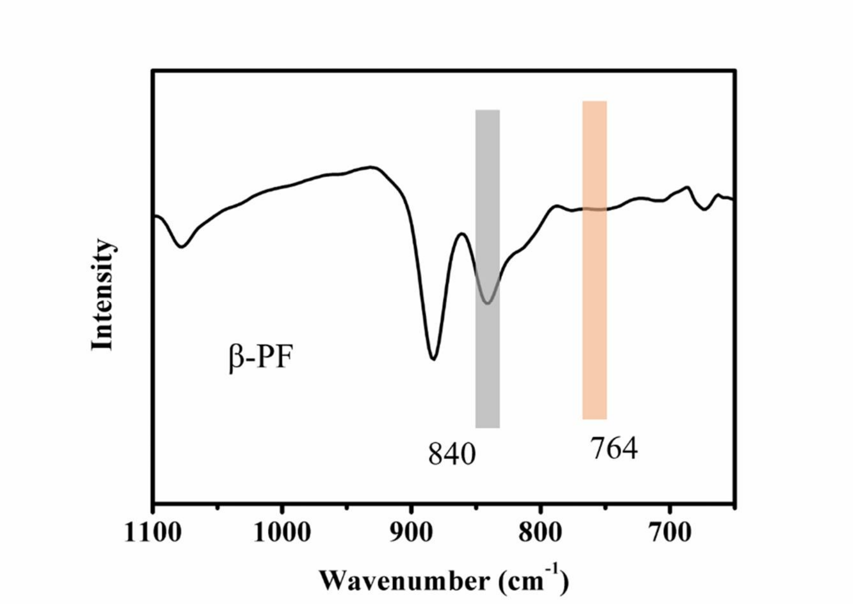

Figure S5. FTIR spectra of β-PF film after cycles.

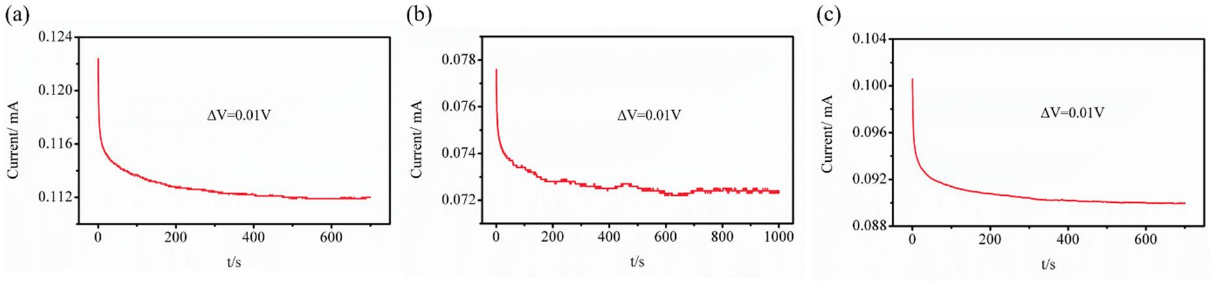

Figure S6. Amperometric i-t curves of traditional 1 M LiTFSI/DOL+DME electrolyte with β-PF@PP/PE (a), re-β-PF@PP/PE (b) and PP/PE (c).

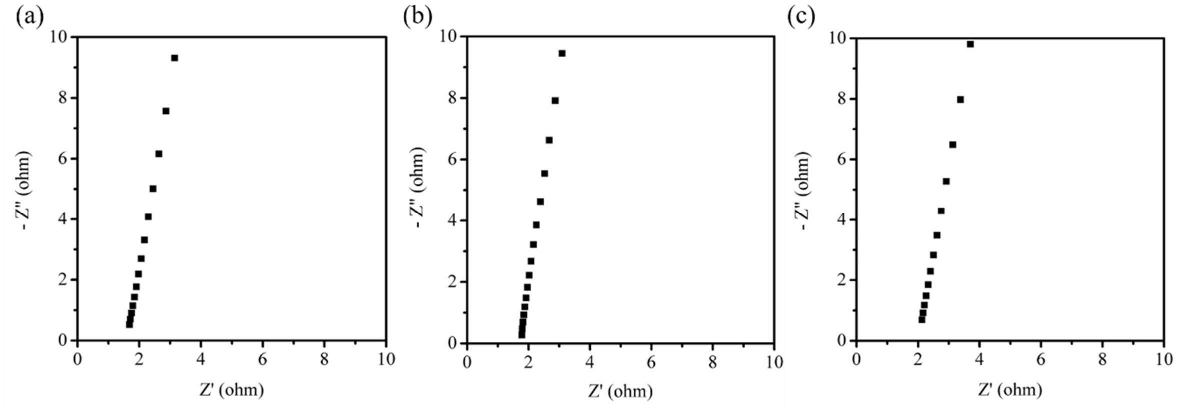

Figure S7. Nyquist plots of the stainless steel | stainless steel cells with β-PF@PP/PE (a), re-β-PF@PP/PE (b) and PP/PE (c).

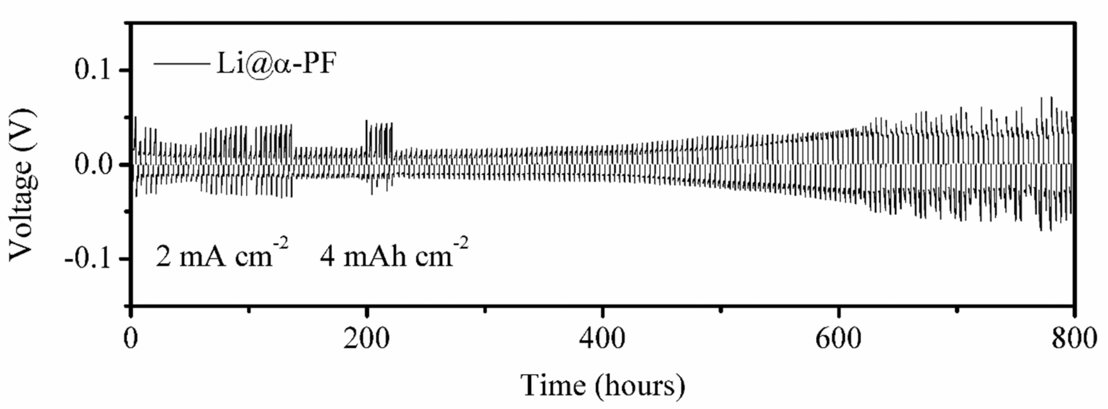

Figure S8. Voltage-time curves of symmetric Li@α-PF | Li@α-PF battery at 2 mA cm^{-2} for 4 mAh cm^{-2}.

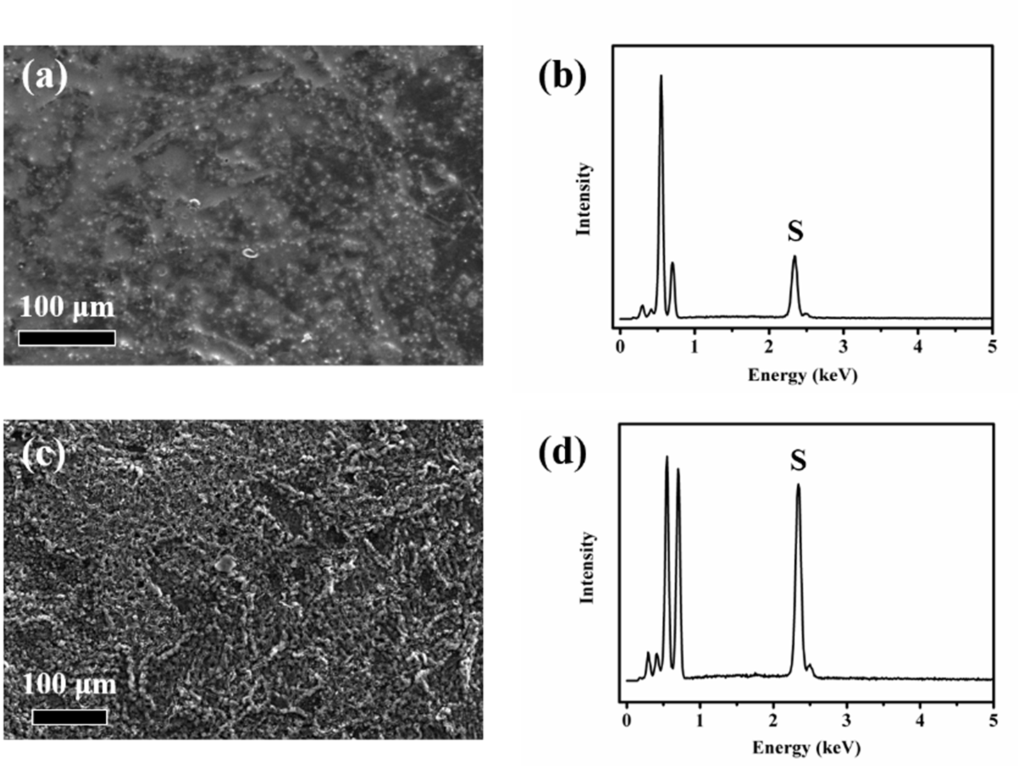

Figure S9. The SEM images and EDS results of Li metal anodes. a, c) SEM images of Li metal anodes with (a) and without (b) β-PF layer modification, b, d) EDS results of Li metal anodes with (b) and without (d) β-PF layer modification.

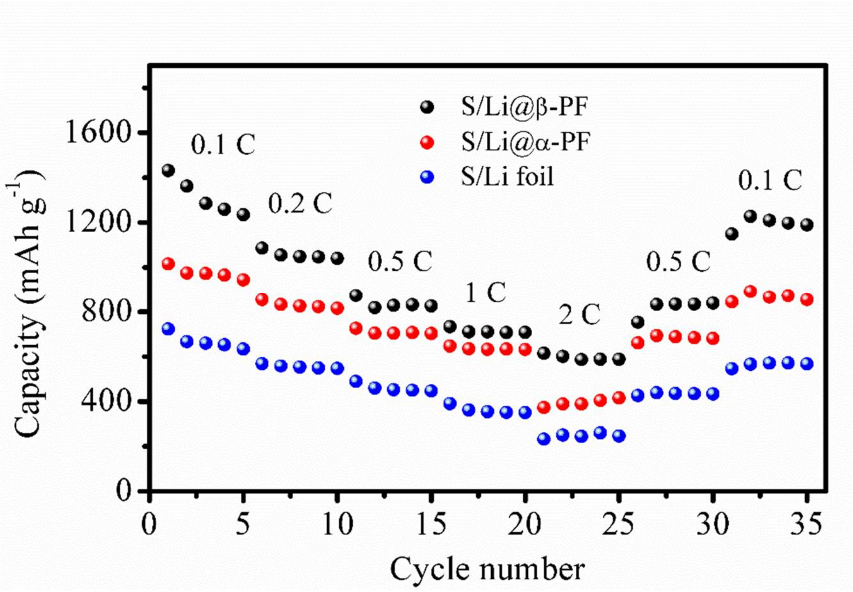

Figure S10. Rate performances of S/Li, S/Li@α-PF and S/Li@β-PF.
